# Naked and Decorated Nanoparticles Containing H_2_S-Releasing Doxorubicin: Preparation, Characterization and Assessment of Their Antitumoral Efficiency on Various Resistant Tumor Cells

**DOI:** 10.3390/ijms231911555

**Published:** 2022-09-30

**Authors:** Elena Peira, Daniela Chirio, Simona Sapino, Konstantin Chegaev, Giulia Chindamo, Iris Chiara Salaroglio, Chiara Riganti, Marina Gallarate

**Affiliations:** 1 Department of Drug Science and Technology, University of Turin, 10125 Turin, Italy; 2 Department of Oncology, University of Torino, Via Santena 5/bis, 10126 Torino, Italy

**Keywords:** doxorubicin derivative, lipid nanoparticles, multi-drug resistance, breast cancer cells, osteosarcoma cells

## Abstract

Several semisynthetic, low-cardiotoxicity doxorubicin (DOXO) conjugated have been extensively described, considering the risk of cytotoxicity loss against resistant tumor cells, which mainly present drug efflux capacity. Doxorubicin 14-[4-(4-phenyl-5-thioxo-5H-[1,2]dithiol-3-yl)]-benzoate (H_2_S-DOXO) was synthetized and tested for its ability to overcome drug resistance with good intracellular accumulation. In this paper, we present a formulation study aimed to develop naked and decorated H_2_S-DOXO-loaded lipid nanoparticles (NPs). NPs prepared by the “cold dilution of microemulsion” method were decorated with hyaluronic acid (HA) to obtain active targeting and characterized for their physicochemical properties, drug entrapment efficiency, long-term stability, and in vitro drug release. Best formulations were tested in vitro on human-sensitive (MCF7) and human/mouse DOXO-resistant (MDA-MDB -231 and JC) breast cancer cells, on human (U-2OS) osteosarcoma cells and DOXO-resistant human/mouse osteosarcoma cells (U-2OS/DX580/K7M2). HA-decoration by HA-cetyltrimethyl ammonium bromide electrostatic interaction on NPs surface was confirmed by Zeta potential and elemental analysis at TEM. NPs had mean diameters lower than 300 nm, 70% H_2_S-DOXO entrapment efficiency, and were stable for almost 28 days. HA-decorated NPs accumulated H_2_S-DOXO in Pgp-expressing cells reducing cell viability. HA-decorated NPs result in the best formulation to increase the inter-cellular H_2_S-DOXO delivery and kill resistant cells, and therefore, as a future perspective, they will be taken into account for further in vivo experiments on tumor animal model.

## 1. Introduction

Doxorubicin (DOXO) is a potent broad-spectrum antineoplastic antibiotic isolated from *Streptomyces* species, which is used alone or in combination with other anticancer drugs in the treatment of hematologic cancers and solid tumors, lymphomas, and sarcomas. It is presently considered one of the most effective chemotherapeutic agents and has been used to treat cancer for over 40 years [1]. The use of DOXO is currently limited to the toxicity it causes to most major organs, especially life-threatening cardiomyopathy.

Over the years, numerous studies were conducted both to synthesize low-toxicity DOXO-conjugates and to develop nanocarriers such as liposomes and nanoparticulate systems, aimed to overcome DOXO life-threatening adverse reactions [2,3].

Many synthetic DOXOs and several semisynthetic DOXO conjugated characterized by lower cardiotoxicity have been extensively described in the literature [4,5,6]. These studies also took into account the risk of loss of their cytotoxicity against tumors with low sensitivity to the drug, which is related to a number of resistance mechanisms, mainly the drug efflux capacity of cancer cells.

With the aim of developing new DOXO derivatives that exhibit less cardiotoxicity and are active against DOXO-resistant tumor cells, some authors [7] synthesized a series of H_2_S-releasing DOXOs (H_2_S-DOXOs) obtained by combining DOXO with appropriate H_2_S donor substructures through an ester bond to C-14. These were found to be less cardiotoxic to H9c2 cardiomyocytes than DOXO, and some of them retained a high level of activity even against DOXO-resistant human sarcoma cell lines, namely U-2OS/DX30 and U-2OS/DX100. The authors suggested that the addition of sulfhydryl groups (SH) may affect the activity of P-glycoprotein (Pgp) by influencing its structure and functionality [8]. Moreover, since H_2_S is a gaseous transmitter, such as NO, it is thought to have cardiovascular protective properties [9,10] and to affect different organs and/or systems. Moreover, its biological activity strongly depends on its concentration; generally speaking, at a high concentration, H_2_S becomes cytotoxic, while at lower concentration, it can have explicit beneficial effects. In previous studies, the same authors have not observed any notable H_2_S-related toxic effects of H_2_S-DOXO [11,12].

Of the various H_2_S-DOXOs developed, doxorubicin 14-[4-(4-phenyl-5-thioxo-5H-[1,2]dithiol-3-yl)]-benzoate (H_2_S-DOXO) was subsequently tested in vitro and in vivo in castration-resistant prostate cancer (CRPC), against which chemotherapy is only transiently effective due to the emergence of chemoresistance, to investigate whether H_2_S-DOXOs are able to overcome drug resistance and to test their safety. In an androgen-independent and DOXO-resistant DU-145 prostate cancer cell line, good intracellular accumulation and high cytotoxicity were observed [13]. The effect of a 3-week injection of H_2_S-DOXO in nude mice previously injected subcutaneously with DU-145 cells was a reduction in tumor volume by 60% and an increase in the percentage of apoptotic cells. In addition, administration of free DOXO was associated with weight loss and cardiotoxicity, whereas H_2_S-DOXO was well tolerated and had a better safety profile. Therefore, the authors demonstrated that H_2_S-DOXO represents novel strategies to reverse chemoresistance in the treatment of CRPC.

Nanotechnology has shown promising advancements in the field of drug development and delivery and its applications for diagnosis, monitoring, control, and treatment of biological systems have been described as nanomedicine [14]. In particular, the exploitation of nanoparticles for tumor treatment and diagnosis has reached such a precision that it can target cancer cells to deliver a drug payload for the treatment [15]. Based on these assumptions, in a subsequent work [16], hyaluronated liposomes containing the same H_2_S-DOXO (HALs-H_2_S-DOXO) were prepared with the aim of exploiting the overexpression in osteosarcomas of the hyaluronic acid (HA) receptor CD44, known to promote tumor growth and metastatic spread [17,18] and whose silencing improves the response to DOXO and other anticancer drugs [19]. HALs-H_2_S-DOXO showed higher in vitro toxicity in mouse DOXO-resistant osteosarcoma cells (K7M2) and human DOXO-sensitive osteosarcoma cells (U-2OS) than DOXO alone or Caelyx^®^. In addition, they exhibited a favorable drug release profile and showed the same cardiotoxicity as the marketed liposomal DOXO Caelyx^®^ in 6-week-old K7M2 tumor-bearing female Balb/C mice. Moreover, neither DOXO nor Caelyx^®^ reduced the growth of these tumors, which were selected as models for CD44- and Pgp-overexpressing osteosarcomas, nor did they induce apoptosis in the tumor, whereas HALs-H_2_S-DOXO increased caspase-3 activation in the tumor and decreased Pgp expression in the tumor.

On the basis of these stimulatory results and of the previous results we obtained with lipophilic DOXO derivatives [20,21], we decided to develop lipid nanoparticles (NPs) in which H_2_S-DOXO should be carried. The idea was to increase H_2_S-DOXO chemical stability towards hydrolysis and to overcome the phenomena of self-aggregation, coalescence, flocculation, and precipitation that can occur during liposome formulation or storage, leading to degradation of the vesicle structure and decomposition of the liposomes themselves [22].

Recently, we developed a NP production technique called “cold dilution of microemulsion” [23,24], which, unlike most methods described in the literature, does not require high temperatures, sonication, or pH fluctuations that would impair drug stability or entrapment. Such technique combines the advantages of an emulsion-solvent diffusion technique with the high stability and super-solvent properties of microemulsion systems. Moreover, H_2_S-DOXO derivatives described in the literature [5] hydrolyzed in human serum due to the presence of two susceptible components in H_2_S-DOXO: the ester group and the H_2_S donor substructure, which follow pseudo-first-order kinetics with t_1/2_ 2.8 h.

We hypothesize that the inclusion of H_2_S-DOXO in a lipid matrix rather than in the aqueous environment of liposomes could improve its stability during storage and after administration.

In this paper, we present a formulation study aimed at selecting the best conditions and the most suitable ingredients for the development of naked and HA-decorated H_2_S-DOXO-loaded NPs. After screening the physicochemical properties, drug entrapment efficiency, long-term stability, and in vitro drug release, the best formulations were tested in vitro on human DOXO-sensitive (MCF7) and DOXO-resistant (MDA-MDB-231) breast cancer cells, on DOXO-resistant mouse breast cancer cells (JC), on human (U-2OS) osteosarcoma cells, DOXO-resistant human osteosarcoma cells (U-2OS/DX580), and on DOXO-resistant mouse osteosarcoma cells (K7M2).

## 2. Results

### 2.1. Solubility and Chemical Stability of H_2_S-DOXO

The solubility of H_2_S-DOXO in aqueous and organic solvents is reported in Table 1.

The chemical stability of H_2_S-DOXO in water/acetonitrile 60/40 *v*/*v* (mobile phase for HPLC analysis) and in methanol (solvent used for extraction from NPs to determining EE%) was tested by RP-HPLC after 30-day storage (t30). In Table 2, the stability was expressed as a percentage of residual concentration at t30 compared with the concentration at t0 (100%).

The chemical stability of H_2_S-DOXO in water/acetonitrile 60/40 *v*/*v* in the presence of µE ingredients, at the same ratio (*w*/*w*) used in the µE, was tested to select the suitable ingredients and to ensure the integrity of H_2_S-DOXO into the formulation. In Table 3, the stability at t0 and t24 h (t24) is expressed as percent of residual concentration in the presence of ingredients compared with that without ingredients at t0 (100%).

The knowledge of the stability and solubility of H_2_S-DOXO in aqueous and organic solvents was essential for the following selection of the ingredients for the NP set-up.

### 2.2. Preparation of NPs

The optimal blank µE formulations (F1 series) without and in the presence of ST or CTAB (2.5% *w*/*w*) are reported in Table 4.

Epikuron ^®^200 and NaGC were selected as surfactant/cosurfactant system. Cremophor ^®^RH60 and NaTC were excluded for H_2_S-DOXO instability problems. ST is an established cationic molecule employed in drug delivery systems to confer a positive charge to the surface of lipid matrix (NPs) [25] or bilayer (liposomes) [26]. ST was selected to make NPs cationic despite the cationic lipid destabilized H_2_S-DOXO, as observed in chemical stability tests (Table 3). In the NP matrix, H_2_S-DOXO could still be protected and not be in direct contact with the positive charge exposed on the surface. NPs were obtained by diluting µEs with water or aqueous solutions of several polymers (PL F68, PVA 9000, and PVP) at different concentrations (1, 2, 3% *w*/*v*).

After the formulation step, it was crucial to determine mean diameters and Zeta potentials of the different NPs.

### 2.3. DLS Analysis of NPs

**Figure 1:**
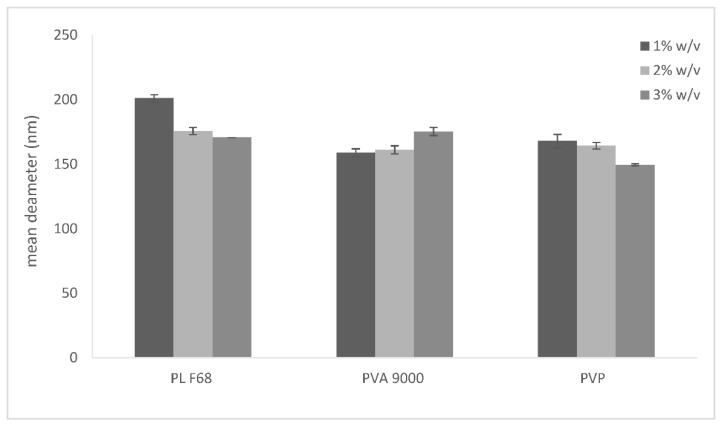
Mean diameters of NPs obtained by dilution of µE F1 with aqueous solutions of several polymers (PL F68, PVA 9000, and PVP) at different concentrations (1, 2, 3% *w*/*v*).

As the mean diameter in water, determined by DLS, was greater than 500 nm, the mean diameters of the NPs (NP series) were determined in aqueous polymer solutions, to choose the best stabilizing polymer (Figure 1).

NPs dispersed in 3% PVP have a mean diameter of around 150 nm, which is the smallest in the whole NP series. A highly negative (around -15 mV, data not shown) Zeta potential was the result for all NP series. The negative surface charge could be due to the presence of NaGC in the lipid matrix, while the disposition of each polymer on NPs, although influencing surface hydrophilicity, does not modify its charge.

On the contrary, when µE F1-ST and F1-CTAB were diluted by water (Table 5), they gave rise to cationic NPs with mean diameters in water (~250 nm) lower than that of NP series. Thus, we may assume that the two amphiphilic molecules stabilize the formulation without the further need of stabilizing polymers.

The presence of a cationic lipid (ST) or a cationic surfactant (CTAB) determined a dramatic change in NP surface charge, as shown by the highly positive value of Zeta potentials, which were in the +18/+20 mV range.

The resulting cationic NPs were decorated with HA via electrostatic interaction between the negative charge of HA and the positive charge of ST or CTAB. The presence of the outer layer of HA dramatically increased particle size and converted the surface charge to a negative value again (Table 6). To obtain HA-decorated NP-ST (HA-NP-ST), NP-ST were dispersed in HA aqueous solutions at different HA concentrations (0.05, 0.1, and 0.2% *w*/*v*). When the HA concentration increased, the negative surface charge also increased.

As the smallest HA-NP-ST were those obtained by dispersing NP-ST in 0.05% *w*/*v* HA, NP-CTAB were also dispersed only in 0.05% *w*/*v* HA (Table 6).

The results of the characterization section allowed us to select NPs with the proper mean diameters and Zeta potentials to be loaded with H_2_S-DOXO.

Indeed, H_2_S-DOXO was added to µE F1 and the formulation was dispersed in 3% *w*/*v* PVP solution (NP-H_2_S-DOXO 3% PVP). H_2_S-DOXO was also incorporated in µEs F1-ST and F1-CTAB and dispersed in water to facilitate the subsequent ionic bond with HA (used at 0.05% w/v). The obtained NPs were characterized by mean diameter, Zeta potential, and encapsulation efficiency (EE%) measurements (Table 7).

A slight increase in the mean diameters in all NPs was noted when compared with empty NPs. Zeta potentials are almost unmodified upon addition of H_2_S-DOXO.

NPs containing ST, as a positively charged agent, have larger diameters than those containing CTAB, and very low EE%, confirming the results obtained from the chemical stability tests of the DOXO-derivative in presence of ST. Therefore, NP-CTAB-H_2_S-DOXO with 0.05% *w*/*v* HA (HA-NP-CTAB-H_2_S-DOXO) or without HA were used for further characterizations, in terms of microscopic observation and stability studies over time.

### 2.4. Optical Microscopy

**Figure 2:**
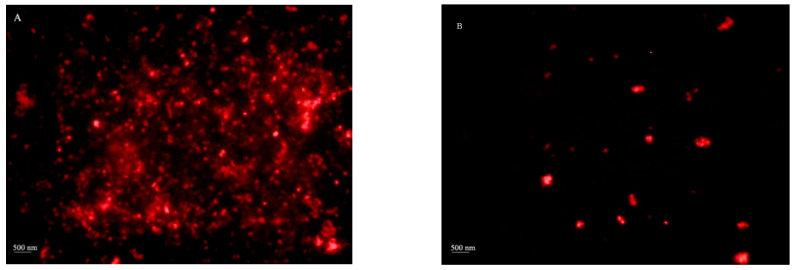
Micrographs of H_2_S-DOXO-loaded NP without HA (NP-H_2_S-DOXO 3% PVP) (**A**) and with 0.05% *w*/*v* HA (HA-NP-CTAB-H_2_S-DOXO) (**B**).

NP-H_2_S-DOXO 3% PVP and HA-NP-CTAB-H_2_S-DOXO microphotographs are reported in Figure 2. The aim of optical microscopy was to detect the presence of a H_2_S-DOXO loaded disperse systems, as only particles with a mean diameter higher than 500 nm were visualized, according to optical microscopy resolution power. Anyway, the presence of H_2_S-DOXO crystals and diffused fluorescence (due to the presence of fluorescent molecule out of the NP matrix) were excluded by observation under polarized light.

### 2.5. In Vitro Drug Release

**Figure 3:**
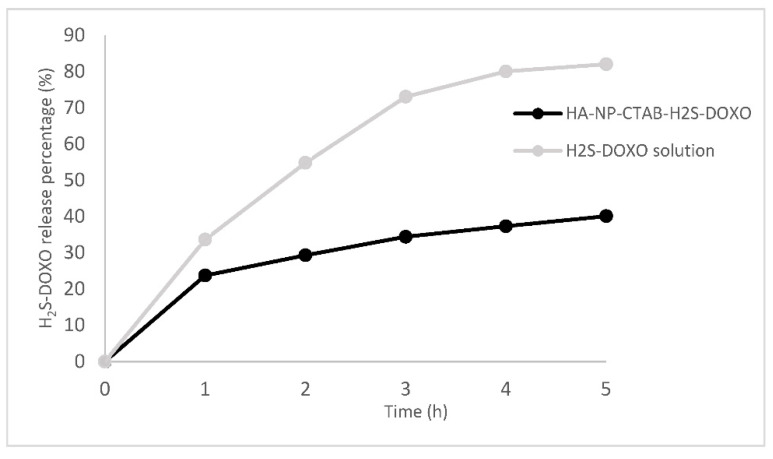
Release profile of H_2_S-DOXO from HA-NP-CTAB-H_2_S-DOXO and in aqueous solution.

Cumulative drug release profiles from HA-NP-CTAB-H_2_S-DOXO and H_2_S-DOXO solution over 5 h are reported in Figure 3.

H_2_S-DOXO release in 5 h was almost 100% from an aqueous solution with a typical first-order curve (R^2^ = 0.999). On the other hand, only 40% of the H_2_S-DOXO incorporated in NP matrix was released after the same time; a typical burst effect was noted in the first hour. These results suggest that most H_2_S-DOXO is likely be entrapped within NPs.

### 2.6. Stability Studies

**Figure 4:**
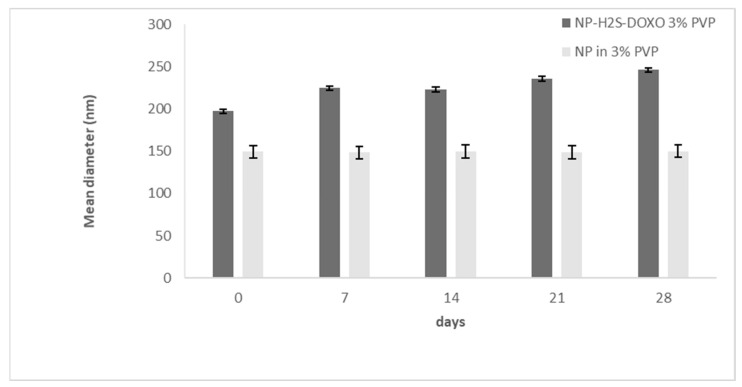
Mean diameter stability of NP-H_2_S-DOXO 3% PVP and NP in 3% PVP over time (28 days).

Stability studies were performed on those systems which had the best characteristics in term of mean diameter, Zeta potential, and EE%. NP-H_2_S-DOXO 3% PVP and the NPs with the same composition but without H_2_S-DOXO (NP in 3% PVP) were stable for 28 days (t28) at 4 °C, as reported in Figure 4. Mean diameters were quite unmodified for blank NP, while a slight, albeit progressive increase was noted in NP-H_2_S-DOXO 3% PVP.

**Figure 5:**
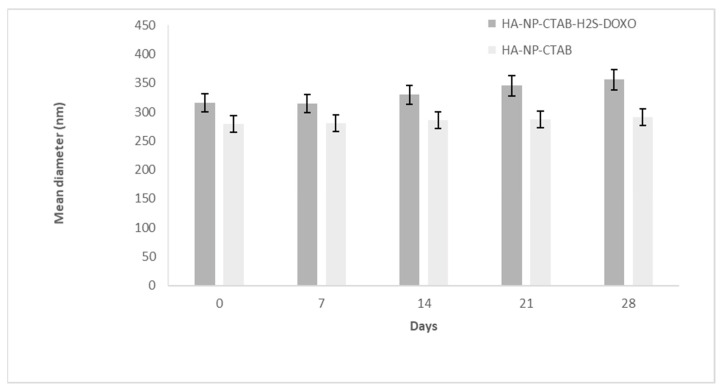
Mean diameter stability of HA-NP-CTAB-H_2_S-DOXO and HA-NP-CTAB over time (28 days).

A similar behavior was noted for HA-NP-CTAB, with and without H_2_S-DOXO (Figure 5).

For NP-H_2_S-DOXO 3% PVP and NP-CTAB-H_2_S-DOXO with and without HA, the Zeta potential remained the same after 28-day storage at 4 °C. In the case of decorated NPs, the stability of the Zeta potential, reported in Table 8, revealed the stability over time of the decoration itself.

The stability of HA-NP-CTAB-H_2_S-DOXO in term of mean diameter, Zeta potential, and EE% was tested at 4 °C and at a controlled room temperature (25 °C) for 72 h, both being only slightly modified, while EE% dropped to 42% at 25 °C storage (Table 9).

### 2.7. TEM Analysis

Morphological analysis by transmission electron microscopy (TEM) was performed on the 0.05% *w*/*v* HA-NP-CTAB. Analyses were carried out on undecorated NP-CTAB, without H_2_S-DOXO, and on HA-NP-CTAB, also without H_2_S-DOXO, since the purpose of the analysis did not require its presence.

TEM observation was mainly aimed to assess the presence of HA on NP surface thanks to elemental analysis. In fact, from this analysis, it was also possible to obtain, in addition to an enlarged image of individual NPs, data regarding the elements present on their surface. In fact, from the morphological analysis, it is difficult to distinguish and discriminate between coated and naked NPs. Thanks to the elemental analysis, however, it was possible to obtain information on the presence or absence of HA.

**Figure 6:**
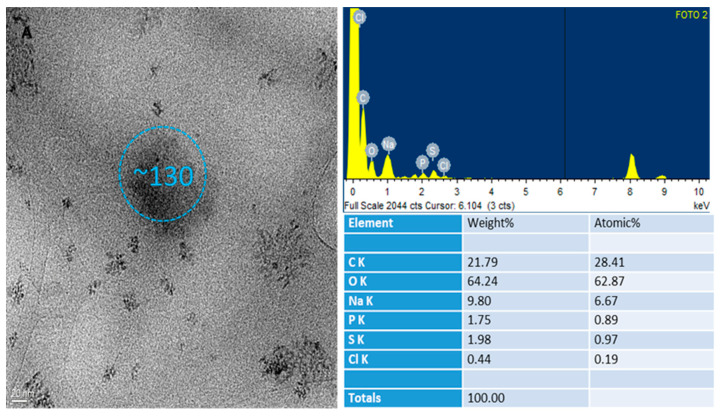
Morphological and elemental analysis of NP-CTAB by TEM.

**Figure 7:**
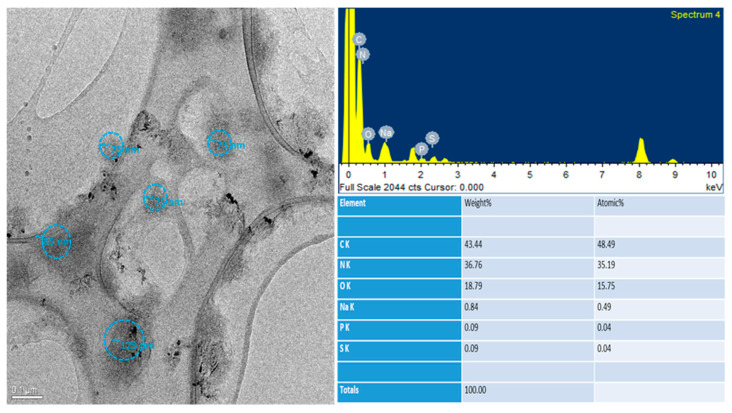
Morphological and elemental analysis of HA-NP-CTAB by TEM.

As shown in Figure 6 and Figure 7, HA-NP-CTAB are small in size and with a morphology similar to the uncoated NPs. We must underline that the single diameter value determined by TEM does not reflect the actual polydisperse situation of NP samples, which, on the other hand, is well defined by DLS analysis. These data in themselves do not give the possibility either to distinguish the two samples or to be able to assert with certainty the presence of the actual surface coating. However, thanks to the atomic spectrum, it was possible to detect the presence of nitrogen atoms in large quantities on the surface of the coated NPs, not highlighted in the atomic spectrum of naked NPs. The presence of nitrogen atoms in the NP indicates the presence of a component that has this chemical element in its molecular structure. Indeed, HA is one of the components used to obtain NP that possesses these characteristics.

Due to the lightness of this chemical element, detecting the presence of nitrogen by TEM is rather difficult when present in traces, as in the case of naked NPs which possess small quantities of CTAB and lecithin (Epikuron ^®^ 200), while its detection on the surface of NPs decorated with HA may be due to the large amount of glycosaminoglycan.

After this deep physico-chemical characterization, the best H_2_S-DOXO NPs were candidate to in vitro studies.

### 2.8. In Vitro Study

#### HA-NP-CTAB-H_2_S-DOXO Increased DOXO Accumulation and Cytotoxicity in Resistant Cells

In a first experimental set, we incubated cells for 6 h with 5 µM free DOXO, free H_2_S-DOXO, NP-H_2_S-DOXO 3% PVP, and HA-NP-CTAB-H_2_S-DOXO (containing 5 µM H_2_S-DOXO).

We chose two tumor types—osteosarcoma and breast cancer—in which DOXO is used as first-line treatment, but where problems of acquired resistance often occur [27,28]. DOXO-sensitive breast cancer MCF-7 cells and DOXO-resistant MDA-MB-231, a triple negative breast cancer cell line [29], DOXO-sensitive osteosarcoma U-2OS cells, and the resistant counterpart U-2OS/DX580 [30] were chosen to compare parental drug-sensitive cells and cells with an acquired resistance to DOXO, a situation that mimics what happens in patients.

**Figure 8:**
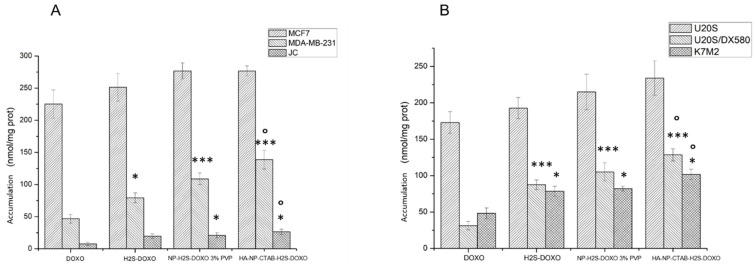
Intracellular DOXO accumulation in: (**A**). breast cancer cells; (**B**). osteosarcoma cells. Cells were incubated 6 h with 5 µM with free DOXO, free H_2_S-DOXO, NP-H_2_S-DOXO 3% PVP, and HA-NP-CTAB-H_2_S-DOXO (containing 5 µM H_2_S-DOXO), then the intracellular accumulation of the drug was measured fluorimetrically (n = 3). Data are means + SD. * *p* < 0.05, *** *p* < 0.001: versus DOXO; ° *p* < 0.05: versus H_2_S-DOXO.

As shown in Figure 8A,B, NP-H_2_S-DOXO 3% PVP did not produce a higher intracellular drug delivery than DOXO or H_2_S-DOXO in sensitive cell lines, characterized by low levels of Pgp [29,30], the main transporter of DOXO. By contrast, NP-H_2_S-DOXO 3% PVP and particularly HA-NP-CTAB-H_2_S-DOXO significantly increased the intracellular drug retention in resistant MDA-MB-231 and U-2OS/DX580 cells, both characterized by high levels of Pgp [29,30]. The same results were obtained in murine JC and K7M2 cells, both characterized by a constitutively very high expression of Pgp and resistance to DOXO [31,32]. Collectively, our results are in line with previous findings indicating that H_2_S-DOXO is less effluxed by Pgp, because it promotes a sulfhydration followed by degradation of the protein, either as free drug [30] or when encapsulated in liposomes [16].

The use of NPs likely offers the further advantage of a better delivery of the drug inside the cells, favoring the accumulation of DOXO. The effect was more pronounced with HA-NP-CTAB-H_2_S-DOXO than with NP-H_2_S-DOXO 3% PVP, likely because HA grants an active targeting of the NPs. Indeed, the HA receptor, CD44, has been detected in U-2OS/DX580 and K7M2 cells [16], and is also expressed in breast cancer as MDA-MB-231 cells [33]. These effects were not species-specific, as the higher accumulation was observed both in human and murine cell lines.

**Figure 9:**
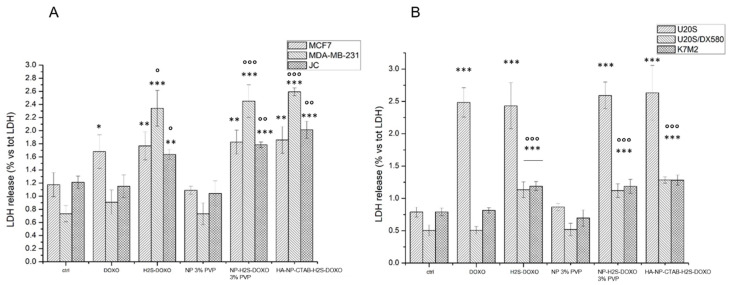
Acute cytotoxicity in: (**A**). breast cancer cells; (**B**). osteosarcoma cells. Cells were incubated 24 h with 5 µM with free DOXO, free H_2_S-DOXO, NP-H_2_S-DOXO 3% PVP, and HA-NP-CTAB-H_2_S-DOXO (containing 5 µM H_2_S-DOXO), then the release of LDH was measured spectrophotometrically (n = 3). Data are means + SD. * *p* < 0.05, ** *p* < 0.01, *** *p* < 0.001: versus ctrl; ° *p* < 0.05, °° *p* < 0.01, °°° *p* < 0.001: versus DOXO.

In line with the different intracellular drug accumulation, the acute toxicity, measured as release of LDH, was comparable between free DOXO, free H_2_S-DOXO, NP-H_2_S-DOXO 3% PVP, and HA-NP-CTAB-H_2_S-DOXO in sensitive MCF-7 and U-2OS cells. By contrast, free DOXO was devoid of any cytotoxic effects against resistant MDA-MB-231, JC, U-2OS/DX580, and K7M2 cells. H_2_S-DOXO, either as free drug or loaded in NPs, induced a significant release of LDH (Figure 9A,B). The toxicity was not caused by NPs themselves, because blank NPs, without H_2_S-DOXO, did not elicited any release of LDH, strongly suggesting the difference in cytotoxicity were due to the NPs’ cargo H_2_S-DOXO. Notably, both H_2_S-DOXO and their NP formulations were more effective than free DOXO against drug-resistant cells. No significant differences were detected between free H_2_S-DOXO, NP-H_2_S-DOXO 3% PVP, and HA-NP-CTAB-H_2_S-DOXO, notwithstanding the higher accumulation of the latter formulations compared to the free drug. This phenomenon can be due to the slower release of H_2_S-DOXO from NP once entered the cells that may provoke a slight delay in the induction of acute damage compared to free H_2_S-DOXO.

**Figure 10:**
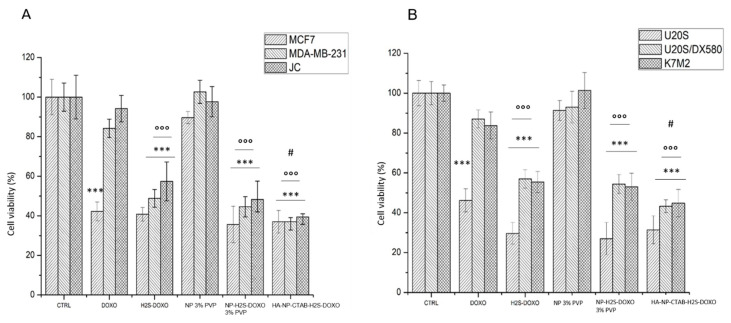
Cell viability: (**A**). breast cancer cells; (**B**). osteosarcoma cells. Cells were incubated 72 h with 5 µM with free DOXO, free H_2_S-DOXO NP-H_2_S-DOXO 3% PVP, and HA-NP-CTAB-H_2_S-DOXO (containing 5 µM H_2_S-DOXO), then the release of LDH was measured by a chemiluminescence-based assay (n = 4). Data are means + SD. *** *p* < 0.001: versus ctrl; °°° *p* < 0.001: versus DOXO; # *p* < 0.05: versus H_2_S-DOXO.

We finally analyzed the subacute toxicity of our formulations, in terms of reduced cell viability. Again, free H_2_S-DOXO, NP-H_2_S-DOXO 3% PVP and HA-NP-CTAB-H_2_S-DOXO did not offer any further advantage compared to DOXO in sensitive MCF-7 and U-2OS cells (Figure 10A,B). However, in contrast, they all reduce cell viability compared to untreated cells in resistant breast and osteosarcoma, of both human and murine origin, where DOXO was ineffective. Free H_2_S-DOXO, NP-H_2_S-DOXO 3% PVP, and HA-NP-CTAB-H_2_S-DOXO were significantly more effective than DOXO against resistant cells. Moreover, the reduction in cell viability elicited by HA-NP-CTAB-H_2_S-DOXO was higher than that elicited by free H_2_S-DOXO, confirming that this formulation was the best performer as anticancer agent, able to increase the intracellular drug delivery and kill resistant cells.

Overall, our data propose the NPs loaded with H_2_S-DOXO offer an excellent strategy to overcome the resistance to DOXO in refractory tumors, because they exploit the intrinsic efficacy of H_2_S-DOXO against Pgp-expressing cells. Moreover, the NP formulation allows a higher drug delivery within tumor cells, due to the endocytosis or active targeting in case of HA-conjugated NPs, and is, thus, superior to free H_2_S-DOXO.

## 3. Materials and Methods

Hyaluronic acid sodium salt 1600 kDa (HA) was purchased from Farmalabor (Barletta, Italy), stearylamine (ST), cetyltrimethylammonium bromide (CTAB), ethyl acetate (EA), propylene glycol, benzyl alcohol, Pluronic^®^F68 (PL F68), polyvinyl alcohol 9000 (PVA 9000), and Kollidon^®^25 (PVP) were from Merck (Darmstadt, Germany), Epikuron^®^200 (phosphatidylcholine 92%) was from Cargill (Minneapolis, MN, USA), doxorubicin hydrochloride (DOXO) was purchased from APAC Pharmaceutical (Columbia, MD, USA), Cremophor^®^RH60 (PEG-60 hydrogenated castor oil) from BASF (Ludwigshafen am Rhein, Germany), taurocholic acid sodium salt (NaTC) and glycocholic acid sodium salt (NaGC) were from ICN Biomedicals (Aurora, OH, USA), and trilaurin and tristearin from Alfa-Aesar (Ward Hill, MA, USA). Deionized water was obtained by a MilliQ water purification system (Millipore, Bedford, MA, USA). Unless specified otherwise, all reagents were purchased from Merck.

### 3.1. H_2_S-DOXO Synthesis Purification and Analysis

The synthesis of H_2_S-DOXO and the RP-HPLC analysis were performed as described in Chegaev et al. [7].

### 3.2. H_2_S-DOXO Solubility in Aqueous and Organic Solvent

The solubility of H_2_S-DOXO in water/acetonitrile (60/40 *v*/*v* ratio), methanol, 0.1 M pH 7.2 phosphate buffer, propylene glycol, and benzyl alcohol was determined as follows: an excess of H_2_S-DOXO was added to the organic or aqueous solution (1 mL); after 2-h equilibration at room temperature (22–23 °C) in a sealed vial under 600 rpm magnetic stirring, the suspension was centrifuged at 1397× *g* for 10 min, and the supernatant solution analyzed by RP-HPLC. The time needed to reach equilibrium solubility was determined by analyzing samples of the equilibrating solution at different time points to establish constant drug solubility.

### 3.3. H_2_S-DOXO Stability in Solvents and in the Presence of Microemulsion Ingredients

H_2_S-DOXO stability in water/acetonitrile (60/40 *v*/*v* ratio) and methanol was determined after 30-day (t30) storage at 4 °C following the drug concentration over time by RP-HPLC and comparing it with the concentration at t0, assuming it as 100%.

H_2_S-DOXO stability in water/acetonitrile (60/40 *v*/*v* ratio) in the presence of NaTC, NaGC, lecithin (phosphatidylcoline 95–98%), ST, CTAB, and Cremophor^®^RH60 was determined at t0 and after 24 h (t24) by RP-HPLC comparing the drug concentration with that in water/acetonitrile solution at t0, assuming it as 100%.

### 3.4. NP Preparation

NPs were prepared by the method named “cold dilution of microemulsion” [24]; briefly, an O/W microemulsion (µE) is prepared, whose disperse phase consists in a solution of a solid lipid dissolved in a partially water miscible solvent. The solvent and the external phase, consisting of phosphate buffer solution (PBS), are mutually saturated at 25 ± 2 °C for 2 h in order to ensure the initial thermodynamic equilibrium of both liquids, before using them in µE formulation. NPs are then obtained by precipitation by quickly diluting µE with a proper volume of aqueous solution to remove the solvent from the dispersed phase and extract it into the continuous phase.

In the present paper, after a preliminary screening on several compositions, µEs were obtained with biocompatible GRAS ingredients (Generally Recognized As Safe) in which H_2_S-DOXO was stable. EA, chosen as water miscible solvent, and the aqueous phase (0.1 M pH 7.2 phosphate buffer) were mutually pre-saturated before using them in µE preparation (EAs and PBSs respectively). A total of 1 mL of the formulated µE was then diluted with 5 mL water or a polymeric aqueous solution to precipitate NPs. Various concentrations of aqueous solutions of several polymers, PL F68, PVA 9000, and PVP, were tested to check the best conditions to obtain small NPs and to avoid NPs aggregation [34]. A very complex and in-depth formulation study was then developed to optimize the NP size.

Finally, 2 mg of H_2_S-DOXO were incorporated in 1 mL of the selected µE, prepared with those surfactants/cosurfactants and cosolvents that allow to maintain the structural drug stability.

H_2_S-DOXO-loaded NPs and free H_2_S-DOXO were separated by gel filtration using Sepharose CL-B4 column.

HA decorated NPs were prepared to obtain active targeting. HA decoration was achieved by the electrostatic interaction between HA and CTAB or ST on the NPs surface [35,36]. CTAB or ST were added in the selected µE. 1 mL of NP-H_2_S-DOXO (containing CTAB or ST and called NP-CTAB-H_2_S-DOXO or NP-ST-H_2_S-DOXO) aqueous dispersion was added into 5 mL of HA solution (0.05, 0.1, 0.2% w/v) dropwise under gentle stirring. After 30 min, electrostatic interactions between HA and cationic NPs led to the formation of HA-NP-CTAB-H_2_S-DOXO or HA-NP-ST-H_2_S-DOXO. Blank HA-NP-CTAB and HA-NP-ST were prepared without H_2_S-DOXO according to the above-described protocol.

Unreacted HA was separated by gel centrifugation using Sephadex^®^G-25 column.

### 3.5. Characterization of NP

*Particle size and Zeta potential measurement.* Particle size and Zeta potential of NPs were determined by dynamic light scattering (DLS) using the Zetasizer Nano ZS90 (Malvern Instruments, UK). Before each measurement, the dispersion of NPs was diluted with Milli-Q water. All measurements were taken at 25 °C and each sample was analyzed in triplicate.

*Optical microscopy analysis.* The possible aggregation of NPs was observed using an optical microscope (LEICA DM 2500 microscope, Leica Microsystems GmbH, Wetzlar, Germany) equipped with a fluorescent lamp (Leica DM 2500, Solms, Germany) connected to a digital camera at × 630 magnification. The microscope images were analyzed using the Motic Images 2000 software.

*Transmission electron microscopy (TEM).* The shape and morphology of naked and HA-decorated NPs were studied by transmission electron microscopy (TEM) via JEOL JEM 3010-UHR (Peabody, MA, USA) microscope operating at 300 kV and equipped with an Oxford Inca Energy TEM 200 EDS X-rays analyzer. For the measurements, samples were prepared by dripping on a carbon-coated copper grid and water was evaporated in air.

*Determination of drug entrapment efficiency (EE%).* H_2_S-DOXO concentration in NP suspension, after elution in gel filtration (GF) and gel centrifugation (GC) column, was determined by the HPLC method used for in vitro quantification of drug. The drug amount resulting after GF and GC was considered as totally entrapped in the NP matrix. EE% is calculated as the ratio between the amount of NP-entrapped drug (post-column drug concentration) and the total amount used in NP preparation (pre-column drug concentration) × 100.

*In vitro drug release.* Release of H_2_S-DOXO from NPs was evaluated using the dialysis method [23]. An aliquot of HA-NP-CTAB-H_2_S-DOXO was placed in a dialysis bag, and the dialysis bag, sealed at the ends, was immersed into 40 mL of phosphate buffered saline (PBS, pH 7.4) at 37 °C stirring at 100 rpm. At predetermined time intervals, 1 mL PBS was withdrawn and replaced with an equal volume of fresh PBS. Release was monitored for 5 h. The overtime released H_2_S-DOXO was analyzed by RP-HPLC. As a reference, a 20% propylene glycol aqueous solution of H_2_S-DOXO was used.

*Stability studies of NP formulations.* The stability of NP systems was studied in terms of mean diameter, Zeta potential, and EE% after 28-day storage at 4 °C and after 72-h storage at 25 °C.

### 3.6. In Vitro Studies

*Cell lines*. Human breast cancer MCF-7 and MDA-MB-231 cells, murine mammary cancer JC cells, human osteosarcoma U-2OS cells, and murine osteosarcoma K7M2 cells were purchased by the American Type Cell Collection (ATCC). U-2OS/DX580 cells were generated by parental U-2OS cells with a stepwise selection in a medium containing increasing concentration of DOXO and after 25 passages maintaining in the presence of 580 ng/mL of DOXO in the culture medium [30]. Cells were grown in DMEM medium with 1% *v*/*v* penicillin-streptomycin and 10% fetal bovine serum, at 37 °C and 5% CO_2_.

In each of the following experimental sets, cells were incubated with specific dilutions of NPs to reach a final concentration of 5 μM H_2_S-DOXO, or with 5 μM DOXO or H_2_S-DOXO, as reference. Samples under study were: free DOXO, free H_2_S-DOXO, NP-H_2_S-DOXO 3% PVP, and HA-NP-CTAB-H_2_S-DOXO.

*Intracellular DOXO accumulation*. 2 × 10^6^ cells were rinsed with fresh medium, detached with trypsin/EDTA (0.05/0.02% *v*/*v*), rinsed in 0.4 mL of a 1:1 solution ethanol/HCl 0.3 N, and sonicated in ice with 10 s (100 W, Sonicator Labsonic, Aubagne, France). Moreover, 5 μL of cell lysate were used to measure the intracellular protein content (BCA1 kit, Sigma-Merck, St. Louis, MO, USA). The remaining sample was used to measure the intracellular content of DOXO fluorimetrically, using a Synergy HTX 96-well reader (Bio-Tek Instruments, Winooski, VT, USA), using λ_em_ = 475 nm, λ_exc_ = 553 nm. The fluorescence units were transformed in nmoles DOXO/mL, according to a titraton curve prepared with scalar dilutions of DOXO (range 1 μM–1 nM). The results were expressed as nmol DOXO/mg cell proteins.

*Acute cytotoxicity.* Culture medium from 2 × 10^6^ cellule was centrifuged at 12,000 *g* for 15 min to pellet cell debris, while cells were washed with PBS twice, detached with trypsin/EDTA (0.05/0.02% *v*/*v*), rinsed in 0.2 mL of 82.3 mM triethanolamine phosphate-HCl (pH 7.6), and sonicated in ice with 10 s (100 W, Sonicator Labsonic); 5 μL of cell lysate was used to measure the intracellular protein content (BCA1 kit). The activity of extracellular lactate dehydrogenase (LDH), considered as an index of DOXO acute cell damage [8], was measured on 50 μL of cell culture medium, incubated at 37 °C with 5 μM NADH and 20 μM pyruvic acid. The same assay was performed on 5 μL of cell lysate, to measure intracellular LDH. The kinetics of NADH oxidation was followed for 6 min measuring the absorbance at 340 nm with a Synergy HTX 96-well reader (Bio-Tek Instruments). The kinetics was linear overtime. Results were expressed as % extracellular LDH/total (extracellular+intracellular) LDH.

*Viability.* 1 × 10^5^ cells were seeded in 96 well plates and incubated for 72 h with DOXO, H_2_S-DOXO or the different NPs. Cell viability was evaluated using the ATPLite kit (PerkinElmer, Waltham, MA), as per manufacturer’s instructions, using a Synergy-HTX 96-well reader (Bio-Tek Instruments, Winooski, VT). The relative luminescence units (RLUs) of the untreated cells were considered 100%; the RLUs of the other experimental conditions were expressed as percentage versus untreated cells.

*Statistical analysis*. All data in the text and figures are provided as means ± SEM. The results were analyzed by a one-way analysis of variance (ANOVA) and Tukey’s test. *p* < 0.05 was considered significant.

## 4. Conclusions

Given the poor solubility of the H_2_S-DOXO in phosphate buffer at pH 7.2, at which the charge on the amino sugar portion is neutralized, H_2_S-DOXO can be more easily internalized into the oily phase of the O/W µE. Its good solubility in the solvents used in the µE formulation improves its loading. µE was formulated using those ingredients in which H_2_S-DOXO was more stable for at least 24 h. Although CTAB was found to be the positively charged agent in which H_2_S-DOXO was most stable, ST was also tested. Unfortunately, subsequently, it was revealed to be not optimal to H_2_S-DOXO loading in NPs. For stability purposes, NPs need to be dispersed in a polymer solution and the 3% *w*/*v* PVP solution was the best in terms of resulting NPs average diameter. Positively charged NPs, stabilized by cationic agents, had optimal diameters when dispersed in water. The successful conjugation of positive NPs with HA was confirmed by the inversion of the Zeta potential charge and by the elemental analysis at TEM. All the developed systems, blank and H_2_S-DOXO-loaded, naked or HA-decorated were stable for at least 28 days in terms of diameters and Zeta potential. Samples must be stored at 4 °C. In an in vitro cell study, H_2_S-DOXO loaded in NPs (more evident in HA-NP-CTAB-H_2_S-DOXO than in NP-H_2_S-DOXO 3% PVP) accumulates in resistant cells with high levels of Pgp more than DOXO and free H_2_S-DOXO, although no increase in acute damage (24 h) was noted, as H_2_S-DOXO was probably released slowly from NPs. Instead, after 72 h, a reduced cell viability was observed in resistant breast and osteosarcoma of both human and murin origin. HA-NP-CTAB-H_2_S-DOXO resulted the best formulation to increase the inter-cellular drug delivery and kill resistant cells.

## Figures and Tables

**Table 1:** H_2_S-DOXO solubility (mg/mL) in aqueous and organic solvents.

Solvents	Solubility (mg/mL)
water/acetonitrile (60/40 *v*/*v*)	3.40 ± 0.50
methanol	3.82 ± 0.30
benzyl alcohol	1.40 ± 0.70
propylene glycol	2.83 ± 0.30
pH 7.2 phosphate buffer (0.1 M)	0.03 ± 0.01

**Table 2:** H_2_S-DOXO stability in water/acetonitrile and in methanol, expressed as a residual concentration percentage.

Solvents	Residual Concentration % t30
water/acetonitrile (60/40 *v*/*v*)	97.3
methanol	98.7

**Table 3:** H_2_S-DOXO stability at t0 and t24 in the presence of µE ingredients, expressed as a residual concentration percentage.

Ingredients	Residual Concentration %	
	t0	t24
NaTC	34	0
NaGC	100	98
Epikuron ^®^200	100	100
Cremophor ^®^RH60	5	0
ST	44	14
CTAB	100	100

**Table 4:** Microemulsion compositions.

Component	F1 (% *w*/*w*)	F1-ST (% *w*/*w*)	F1-CTAB (% *w*/*w*)
Trilaurin	3.90	3.81	3.81
Tristearin	0.43	0.42	0.42
EA_s_	13.02	12.70	12.70
Epikuron ^®^200	14.47	14.11	14.11
ST	-	2.50	-
CTAB	-	-	2.50
NaGC	5.06	4.94	4.94
PBS_s_	50.62	49.37	49.37
Benzyl alcohol	6.43	6.27	6.27
Propylene glycol	6.02	5.87	5.87

**Table 5:** Mean diameters and Zeta potential values of NP-ST and NP-CTAB.

NPs	Mean Diameter (nm) ± S.E.	I.P	Ζeta Potential (mV) ± S.E.
NP-ST	250.5 ± 9.5	0.159	+18.3 ± 1.3
NP-CTAB	245.0 ± 12.8	0.202	+19.8 ± 1.8

**Table 6:** Mean diameters and Zeta potentials of HA-NP-ST and HA-NP-CTAB.

NPs	Mean Diameter (nm) ± S.E.	I.P	Zeta Potential (mV) ± S.E.
0.2% HA-NP-ST	962.6 ± 91.0	0.399	−21.27 ± 0.14
0.1% HA-NP-ST	759.0 ± 82.4	0.415	−19.21 ± 0.32
0.05% HA-NP-ST	389.9 ± 36.6	0.172	−18.12 ± 0.20
0.05% HA-NP-CTAB	279.3 ± 34.8	0.283	−20.05 ± 0.82

**Table 7:** Mean diameters, Zeta potentials, and EE% of H_2_S-DOXO-loaded NPs.

NPs	Mean Diameter (nm) ± S.E.	I.P	Zeta Potential (mV) ± S.E.	EE%
NP-H_2_S-DOXO 3% PVP	197.2 ± 7.9	0.258	−15.90 ± 1.99	71
NP-ST-H_2_S-DOXO	287.6 ± 13.9	0.203	+21.30 ± 2.14	22
NP-CTAB-H_2_S-DOXO	274.9 ± 14.3	0.144	+21.81 ± 0.76	71
HA-NP-ST-H_2_S-DOXO	443.7 ± 13.7	0.297	−22.55 ± 2.70	21
HA-NP-CTAB-H_2_S-DOXO	307.0 ± 43.3	0.246	−23.50 ± 3.04	70

**Table 8:** Zeta potential of NP-H_2_S-DOXO 3% PVP, NP-CTAB-H_2_S-DOXO, and HA-NP-CTAB-H_2_S-DOXO over time (28 days).

Time (Days)	Zeta Potential (mV) of NP-H_2_S-DOXO 3% PVP ± S.E.	Zeta Potential (mV) of NP-CTAB-H_2_S-DOXO± S.E.	Zeta Potential (mV) of HA-NP-CTAB-H_2_S-DOXO ± S.E.
0	−15.90 ± 1.90	+21.81 ± 0.76	−23.50 ± 3.04
7	−38.65 ± 1.10	+20.61 ± 0.69	−20.67 ± 1.02
14	−36.26 ± 4.72	+21.10 ± 0.60	−21.70 ± 2.06
21	−37.40 ± 1.22	+21.31 ± 0.91	−20.83 ± 2.03
28	−33.36 ± 1.91	+20.98 ± 0.36	−20.56 ± 1.71

**Table 9:** Mean diameter, Zeta potential, and EE% of HA-NP-CTAB-H_2_S-DOXO after 72 h of storage at 4 °C and at controlled room temperature (25 °C).

Temperature	4 °C	25 °C
Mean diameter (nm) ± S.E.	310.0 ± 14.8	374.7 ± 9.5
Zeta potential (mV) ± S.E.	−21.50 ± 2.10	−24.08 ± 3.20
EE%	70	42

## Data Availability

Not applicable.
